# Supplementary material for: The Systems Biology Research Tool: evolvable open-source software
Source: BMC Syst Biol. 2008 Jun 29;2:55. doi: 10.1186/1752-0509-2-55 (PMC2446383; doi:10.1186/1752-0509-2-55)
Supplement: Additional file 1 — SBRT Archive. An archive of the current version of the Systems Biology Research Tool. [file 1752-0509-2-55-S1.zip › sbrt-1.4.0/doc/developers_guide/api/sbrt/kernel/fba/class-use/SinkRxn.html]

Uses of Class sbrt.kernel.fba.SinkRxn


|  |  |  |
| --- | --- | --- |
| **Overview** \| **Package** \| **Class** \| **Use** \| **Tree** \| **Deprecated** \| **Index** \| **Help** | |  |
| PREV   NEXT | **FRAMES**    **NO FRAMES**     **All Classes** |  |


---


## **Uses of Class sbrt.kernel.fba.SinkRxn**

| Packages that use SinkRxn | |
| --- | --- |
| **sbrt.kernel.fba** | Provides classes and interfaces for creating objects relevant to Flux Balance Analysis. |
| **sbrt.shell.text.fba** | Provides classes and interfaces for defining formats for objects relevant to Flux Balance Analysis. |

Uses of SinkRxn in sbrt.kernel.fba

| Methods in sbrt.kernel.fba that return SinkRxn | |
| --- | --- |
| `SinkRxn` | `IrrevRxnFluxome.getSinkRxn(java.lang.String rxnName)`             Returns the sink reaction in this fluxome with the provided name. |
| `SinkRxn` | `IrrevRxnFluxomeV1.getSinkRxn(java.lang.String reactionName)`             Returns the sink reaction in this fluxome with the provided name. |

| Methods in sbrt.kernel.fba that return types with arguments of type SinkRxn | |
| --- | --- |
| `java.util.Map<java.lang.String,SinkRxn>` | `IrrevRxnFluxome.getSinkRxns()`             Returns the set of all sink reactions in this fluxome. |
| `java.util.Map<java.lang.String,SinkRxn>` | `IrrevRxnFluxomeV1.getSinkRxns()`             Returns all of the sinks in this fluxome. |
| `java.util.Map<java.lang.String,SinkRxn>` | `IrrevRxnFluxome.getSinkRxns(java.util.Collection<java.lang.String> rxnNames)`             Returns the sink reactions with the provided names. |
| `java.util.Map<java.lang.String,SinkRxn>` | `IrrevRxnFluxomeV1.getSinkRxns(java.util.Collection<java.lang.String> rxnNames)`             Returns the sink reactions whose names appear in the provided collection of reaction names. |

Constructor parameters in sbrt.kernel.fba with type arguments of type SinkRxn

`IrrevRxnFluxomeV1(java.util.Map<java.lang.String,IrrevRxn> internalRxns,
java.util.Map<java.lang.String,SourceRxn> sourceRxns,
java.util.Map<java.lang.String,SinkRxn> sinkRxns)`
  
          Constructs a new irreversible reaction fluxome from the
provided internal and exchange reactions.

`IrrevRxnFluxomeV1(java.util.Map<java.lang.String,IrrevRxn> internalRxns,
java.util.Map<java.lang.String,SourceRxn> sourceRxns,
java.util.Map<java.lang.String,SinkRxn> sinkRxns,
java.util.Map<java.lang.String,Or<And<java.lang.String>>> catalysts)`
  
          Constructs a new irreversible reaction fluxome from the
provided internal and exchange reactions and catalysts.

Uses of SinkRxn in sbrt.shell.text.fba

| Methods in sbrt.shell.text.fba with parameters of type SinkRxn | |
| --- | --- |
| `java.lang.String` | `IrrevRxnFormatV1.format(SinkRxn sink)`             Returns a formatted string representation of the provided sink. |

---


|  |  |  |
| --- | --- | --- |
| **Overview** \| **Package** \| **Class** \| **Use** \| **Tree** \| **Deprecated** \| **Index** \| **Help** | |  |
| PREV   NEXT | **FRAMES**    **NO FRAMES**     **All Classes** |  |


---
